# Mitochondrial Ataxias: Molecular Classification and Clinical Heterogeneity

**DOI:** 10.3390/neurolint14020028

**Published:** 2022-04-02

**Authors:** Piervito Lopriore, Valentina Ricciarini, Gabriele Siciliano, Michelangelo Mancuso, Vincenzo Montano

**Affiliations:** Neurological Institute, Department of Clinical and Experimental Medicine, University of Pisa, 56126 Pisa, Italy; piervito.lopriore@gmail.com (P.L.); valentinaricciarini2@gmail.com (V.R.); gabriele.siciliano@unipi.it (G.S.); v.montano89@gmail.com (V.M.)

**Keywords:** ataxia, mitochondrial diseases, MERRF, NARP, Kearns-Sayre syndrome, *POLG1*-related ataxia

## Abstract

Ataxia is increasingly being recognized as a cardinal manifestation in primary mitochondrial diseases (PMDs) in both paediatric and adult patients. It can be caused by disruption of cerebellar nuclei or fibres, its connection with the brainstem, or spinal and peripheral lesions leading to proprioceptive loss. Despite mitochondrial ataxias having no specific defining features, they should be included in hereditary ataxias differential diagnosis, given the high prevalence of PMDs. This review focuses on the clinical and neuropathological features and genetic background of PMDs in which ataxia is a prominent manifestation.

## 1. Introduction

Primary mitochondrial diseases (PMDs) are a group of heterogeneous diseases, characterised by defects in any of the multiple mitochondrial metabolic pathways, despite being traditionally associated with defects of the mitochondrial respiratory chain, site of oxidative phosphorylation (OXPHOS) [1].

PMDs are the most frequent metabolic disorders in humans, with a prevalence of approximately 1 in 4300 cases [2]. They can be caused by mutations in mitochondrial DNA (mtDNA) or nuclear DNA (nDNA) displaying maternal and Mendelian inheritance patterns. For both nDNA and mtDNA pathogenic variants can also occur de novo [3]. mtDNA encodes 13 respiratory complex subunits and several RNA elements of the mitochondrial translation system, whereas nDNA encodes the majority of the respiratory complex subunits and mitochondrial expression and replication apparatus. The unique features of mitochondrial genetics explain the clinical heterogeneity of PMDs and the resulting diagnostic challenges encountered by physicians [4]. PMDs may manifest in childhood or adulthood with symptoms affecting a single organ or multiple organ systems. PMDs show a predilection for high-energy demanding tissues, such as the peripheral nervous system (PNS), central nervous system (CNS), eyes, inner ears, heart, gastro-intestinal system, endocrine system, kidneys, and bone marrow [5]. In mitochondrial medicine, the debate between ‘splitters’, who assigned acronyms to well-defined syndromic manifestations, and ‘lumpers’, who consider individual clinical phenotypes as variations of a continuum, is still unsettled. Overlapping syndromes, however, seem to be exceptions rather than rules [4].

Within the CNS pathological manifestations of PMDs, movement disorders, either hypo- or hyperkinetic, have been reported. The relative incidence, features and spectrum of these conditions are still largely unknown and are currently being investigated [6] A recent clinical cross-sectional investigation on an Italian cohort of individuals with childhood-onset PMDs, reported that a movement disorder was present at the onset of 40% of patients, appearing on average five years before it [7]. In adults, parkinsonism is often described, whereas, in the paediatric population, chorea and dystonia are common manifestations. Pure cerebellar, sensitive, or spinocerebellar ataxia, is widely observed in both paediatric and adult PMDs patients, approximating classical hereditary ataxias onset if present as a prominent syndromic aspect [6,8]. The clinical and genetic spectrum of hereditary ataxias, a group of neurological disorders characterised by the slow progressive incoordination of gait in a variable association with speech, eye, and fine movements poor coordination, is heterogeneous. They may result from the cerebellar system or spinal cord dysfunction, or from a combination of both and can be inherited in autosomal dominant, autosomal recessive, X-linked and maternal manner [9]. Mitochondrial ataxias are genetically variegated and are associated with both mtDNA and nDNA defects. They may be part of specific syndromic or non-syndromic PMDs as consequences of genetic defects directly impairing OXPHOS activity, or due to defects in nuclear genes leading to secondary energetic dysfunction.

In this review, we will describe the principal PMD phenotypes associated with ataxia, by using the molecular classification of PMDs as a reference.

## 2. Molecular Classification of Mitochondrial Disorders and Pathogenesis

PMDs classification, initially clinical and biochemical based, is currently built on genetic evidence; PMDs can be caused by mtDNA mutations or defects in nDNA genes functionally relevant for OXPHOS. A more feasible way to characterise them, however, is considering a third class of diseases, caused by abnormalities of genes involved in mtDNA maintenance: defects of intergenomic communication indeed, can cause secondary alteration in mtDNA [3]. 

Human mtDNA is a 16.5 kilobase (kb) circular minichromosome, made up of two different strands (the heavy and light strand), encoding 13 of the 85 (or more) polypeptides that compose the respiratory chain located in the mitochondrial internal membrane (IMM). mtDNA encodes also two ribosomal RNAs (rRNA) and 22 transfer RNAs (tRNA) [5]. The complexity of mitochondrial genetics is more easily comprehensible by considering three essential rules: (1) mtDNA molecules are present in multiple copies within a single cell which can be identical (homoplasmy) or not (heteroplasmy); therefore, the clinical manifestations of an mtDNA-related disease depend largely on the relative proportion between normal and mutant mtDNA variants (threshold effect). (2) During cell division, mtDNAs stochastically distribute among daughter cells contributing to shifting the single-cell mutation load. This phenomenon may explain phenotype changes as disease progresses with age. (3) All zygotic mtDNA comes from the oocyte, thus mtDNA defects follow a maternal inheritance pattern [4]. Moreover, it is worth remembering that mtDNA abnormalities can be point mutations or large-scale rearrangements (single deletions or duplications); the former being homoplasmic or exclusively heteroplasmic and maternally inherited, the latter often recognised as heteroplasmic and sporadic [3]. As molecular research progresses, numerous elements complicating this simplistic view have been recognised, such as the discoveries of putatively ‘neutral’ polymorphisms (usually being homoplasmic) [10] and low levels of heteroplasmy in healthy individuals [11], or the evidence of nuclear and mitochondrial factors influencing the stereotypical phenotypic expression of homoplasmic mutations (often restricted to a single tissue). Their description, however, is not the aim of this review.

Highly adapted to the cellular environment through an endosymbiotic link, mitochondria have almost completely lost their genomic autonomy, depending on nDNA for the codification of most of the respiratory chain subunits, their assembling factors and the synthesis of IMM phospholipids [12]. Moreover, mtDNA replication and translation are controlled by nDNA factors [4]. Whole-exome sequencing studies have made possible a sub-classification of mendelian PMDs based on the specific component of mitochondrial biology affected: (1) respiratory chain subunits (direct hits), ancillary proteins (indirect hits) and electron carriers, (2) mtDNA replication and expression, (3) mitochondrial dynamics, homeostasis and quality control, (4) metabolism of substrates, (5) metabolism of cofactors, (6) toxic compounds metabolism, (7) other functions [13]. Finally, as previously mentioned, abnormalities of nuclear genes involved in mtDNA replication and expression system (group 2) are considered separately; dysfunctions of the intergenomic flow of information, combine features of mendelian and mitochondrial genetics. These disorders are characterised by multiple mtDNA deletions, mtDNA depletion and site-specific mtDNA point mutations. From a molecular perspective, they can be mainly due to defects in the replication apparatus or in the mitochondrial deoxynucleoside triphosphates (dNTPs) pool [13]. However, it is important to underlie that some genes belonging to the above-mentioned groups are controversially disputed as the cause of PMDs, since not all are physically located within the mitochondria or perturb OXPHOS functioning [13]. Despite this in-depth knowledge of PMDs genetic aetiology, our understanding of their pathophysiology is very limited. Pathogenic variants in approximately 413 nuclear and mitochondrial genes have been ascribed as actual or putative causes of PMDs but genotype-phenotype correlations, even for clinically defined syndromes, are insufficient [13]. For example, Leigh syndrome (LS) is associated with defects in more than 90 genes [14], as well as different syndromes that correlate with m.3243A > G mutation in *MT-TL1* (genetic pleiotropy) [15]. Undoubtedly, impaired ATP synthesis has an important pathogenic role. Nevertheless, considering that mitochondria are crucial for such variegated cell life and death functions, including generation of reactive oxygen species (ROS), calcium homeostasis and regulation of apoptosis, it is likely to assume PMDs pathophysiology involves a different extent all of these [3]. Genetic defects that do not directly disrupt OXPHOS activity but impact these mitochondrial functions, cause chronic clinical phenotypes, rather than acute forms of encephalomyopathies. These phenotypes resemble the clinical appearance of the most recurring neurodegenerative disorders (Parkinson’s disease, Alzheimer’s disease, amyotrophic lateral sclerosis), in which mitochondrial dysfunction plays crucial pathogenic roles [4,16]. 

By moving further into the genomic era, the traditional biochemical definition of PMDs has been challenged; however, to date, the lack of knowledge regarding PMDs pathophysiology and the absence of uniform criteria to establish a gene or a variant as causative, complicate their definition.

## 3. Diagnosis of Mitochondrial Disorders

Because of the vast clinical and genetic variability, diagnosing PMDs can be challenging [17].

Clinical assessment is the first step of the PMDs diagnostic pathway and requires a careful evaluation of family history and a fine assessment of organ involvement. Given the peculiarity of mitochondrial genetics, maternal inheritance is indicative of an mtDNA-related disorder, however, heteroplasmy could mask it. Parental consanguinity instead, suggests autosomal recessive inheritance. Sometimes, the misevaluation of family ‘soft signs’, such as hearing loss, fatigue, migraine or exercise intolerance, may not raise suspicion of a genetic disorder. Moreover, it is worth remembering that both nDNA and mtDNA pathogenic variants can occur de novo; mtDNA deletions, for example, are usually sporadic [18]. In mtDNA-related PMDs the onset is variable, even within the same family; the degree of heteroplasmy, however, has been shown to correlated with disease severity and age at onset, in some specific mitochondrial syndromes [19]. On the contrary, nDNA-related PMDs, with the exception of mtDNA maintenance disorders in which adults with progressive external ophthalmoplegia (PEO) represent the most common phenotype, occur in infancy or early childhood [3]. Mitochondria play a cardinal role in cellular energy generation, thus PMDs frequently affect high-energy-demanding tissues, especially CNS, PNS and muscle tissue. They can present as multisystem disorders or more rarely affect single tissue (pure myopathy or cardiomyopathy). Other post-mitotic tissues, such as the heart, the retina and endocrine glands may also be affected. Table 1 includes an extensive list of PMD’s clinical features [5]. Phenotypic expression varies depending on onset age. Adults usually manifest signs of myopathy in association with variable PNS and CNS involvement; some adults may manifest only muscle weakness and exercise intolerance. Infants frequently exhibit psychomotor delay, hypotonia, lactic acidosis and cardiorespiratory failure in the context of fatal complex multisystem disorders, severe encephalomyopathies or isolated myopathies occasionally accompanied by cardiopathies [3,5].

In addition to clinical assessment, routine laboratory investigations and specific serum and urine biomarker assays should be performed to exclude mimics and stratify the population, estimating the likelihood of PMD. FGF-21 and GDF-15 offer higher utility than traditional lactate, pyruvate and creatine-kinase blood assay [20]. A recent meta-analysis comprehensively examined randomized controlled clinical trials to reinvestigate the diagnostic accuracy of FGF-21 and GDF-15 for PMDs. FGF-21 and GDF-15 showed a pooled sensitivity and specificity of 0.71, 0.88 and 0.83, 0.92, respectively [21]. Neuroimaging, nerve conduction studies, electromyography, cardiac and retinal evaluation may support the diagnosis [22,23].

Since Luft’s first reported case of PMD in 1962 [24], biochemical, enzymatic and histopathological evidence of OXPHOS impairment from muscle biopsies has dominated the diagnostic scene, setting up the traditional biopsy-first approach [25]. Compared to Sanger mtDNA sequencing on muscle samples [26], the association of mtDNA sequencing with targeted NGS panels on nuclear genes, and more recently, with whole-exome sequencing, further improves the diagnostic capacity (to between 6–37% and 35–68%, respectively) [27,28,29]. Moreover, in recent years, a less invasive, genetic-first approach based on early bigenomic WGS in stratified cases has been proposed [29].

## 4. Hereditary Ataxias

Ataxia, from Greek ‘lack of order’, is a common manifestation of different neurological conditions characterised by motor and balance incoordination [30]. Cerebellar ataxia is caused by disorders affecting the cerebellum and its connections. It is characterised by the loss of balance with an unsteady and irregular wide-based gait, swaying, increased risk of falls and irregular, fragmented and tremulous limb movements. Nystagmus, uncalibrated eye movements and slurred speech with speed and volume impairment are additional manifestations. Sensory ataxia affects patients with significant proprioceptive loss, caused by spinal or peripheral lesions. It is characterised by a stepping gait that worsens with loss of visual fixation [30]. Spinocerebellar ataxia results from disorders affecting both proprioceptive and cerebellar systems, manifesting as a combination of cerebellar and sensory ataxias.

Ataxias can be acquired/degenerative or inherited. Hereditary ataxias have a prevalence of 8.9/100,000 and can be congenital, episodic or more frequently, progressive [31]. If not ‘pure’, they may be associated with additional features (i.e., ophthalmoplegia, pigmentary maculopathy, spasticity, neuropathy, cognitive impairment) [9]. Hereditary ataxias are generally classified according to the mode of inheritance (AD, AR, XL, maternal trait).

Autosomal dominant cerebellar ataxias (ADCAs) or more generically spinocerebellar ataxias (SCAs), have a variable onset, generally in adulthood, and a variable phenotypic expression usually progressing over decades; juvenile-onset forms are typically more aggressive [32,33]. Mutations associated with ADCAs include: short tandem repeats (STRs) in the coding region, non-coding expansion and less frequently, conventional mutations [9].

Autosomal recessive cerebellar ataxias (ARCAs) are slow progressive ataxia, with an early onset, occurring before twenty years of age. The most common ARCAs are: Friedreich ataxia (FRDA) (estimated prevalence 2–4/100,000), ataxia-telangiectasia and FRDA with atypical presentation (estimated prevalence 1/100,000) [9,34].

X-linked hereditary ataxias (XLCAs) are an expanding group of genetically determined ataxias often associated with cerebellar dysgenesis (hypoplasia, dysplasia or atrophy) [35]. The pathogenesis of some of the above-described hereditary ataxias has a well-known secondary mitochondrial involvement. Defects in genes associated with mitochondrial iron homeostasis, such as *Frataxin* and *ABC7*, cause secondary OXPHOS dysfunction and are related to FRDA and X-linked sideroblastic anaemia and ataxia (XLSA/A), respectively [9].

Maternally inherited ataxias, due to mtDNA mutations, will be deeply discussed in the next paragraph.

Establishing the diagnosis of hereditary ataxias requires a three-generation family history collection and an accurate neurological examination. Note that late-onset sporadic genetic ataxias may be misdiagnosed as non-genetic forms. Acquired causes must be ruled out [31].

Brain MRI often shows atrophy with both ‘pure’ cerebellar or more severe combined cerebellar/brainstem patterns at different grades [36].

In the case of a dominant family history, first-line genetic screening includes SCA 1, 2, 3, 6, 8, 17 and dentatorubral-pallidoluysian atrophy (in Asians). In the case of a suspected recessive disease, it is recommended to first screen for FRDA, ataxia-telangiectasia, spastic ataxias, *POLG1* mutations and oculomotor apraxia type 1 and 2. In sporadic cases, the probability of a genetic cause, including the most common SCAs and FRDA, amounted to 13%; in these cases, multiple system atrophy should be considered for differential diagnosis [9,37].

Although NGS majorly contributes to new pathogenic variant discoveries, some technical limitations prevent the adoption of NGS gene panels in hereditary ataxia clinical practice and the replacement of traditional low-throughout molecular tests for STRs detection [38].

## 5. Mitochondrial Ataxias

The widespread neurological involvement in PMDs is intuitively explained considering the significant sensitivity of neurons to fluctuations in mitochondria energy production. The Purkinje cells of the cerebellum are among the higher energy-demanding cells in the human body, thus particularly vulnerable to mitochondrial dysfunction [39]. In post-mortem brain samples from patients harbouring mtDNA defects, evidence of synaptic alterations in Purkinje connections have been documented; a direct relationship between cellular respiratory deficiency and cerebellar cell density in the same samples has been revealed [39]. Furthermore, microangiopathy seems to be one of the multiple mechanisms likely contributing to ataxia pathophysiology in mtDNA related-PMDs [40].

Recent studies on a childhood-onset PMDs cohort reported ataxia being the most common movement disorder at onset (49% of individuals) [7]. Furthermore, ataxia and parkinsonism were the most represented movement disorders in a large cohort of mitochondrial patients from an Italian registry, with an overall prevalence at last follow-up of 59.1% and 30.5%, respectively [8]. However, no specific features define mitochondrial ataxia, except the common co-occurrence with the above-mentioned neurological and non-neurological signs and symptoms of PMDs (Table 1). In PMDs, cerebellar ataxia is often progressive, from gait and lower limbs to arms; in some cases, a stepwise pattern of progression reflecting a stroke-like evolution can occur [31]. Sensory ataxia is a less common feature in PMDs and might suggest specific syndromes such as *POLG1*-related neuropathy and neuropathy, ataxia and retinitis pigmentosa syndrome (NARP) [41]. Lastly, intermittent ataxia has been described as a genetic defect of the E1 component of the pyruvate dehydrogenase complex [42].

As for most classical hereditary ataxias, brain MRI commonly exhibits different degrees of midline or hemisphere cerebellar atrophy. However, peculiar alterations may be searched, for mitochondrial ataxia recognition or to support the diagnosis of PMDs (see below).

Guided by the molecular classification, we will describe the main clinical, genetic and histopathological features of PMDs, either due to defects in mtDNA (Table 2) or nDNA (Table 3), in which ataxia can occur as a common neurological manifestation. Note that in Table 2 and Table 3, the division between cerebellar, sensory and spinocerebellar ataxia has a didactic aim. Nevertheless, PMDs are marked by a high variability of phenotypic expression; despite some disorders being characterised by a peculiar type of ataxia at onset, overlapping phenotypes are frequent.

### 5.1. Disorders of mtDNA Defects: Point Mutations

#### 5.1.1. Myoclonic Epilepsy and Ragged Red Fibers (MERRF)

MERRF is a multisystem disorder, with a typical onset in childhood, characterised by myoclonus, generalized seizures, muscle weakness and dementia. Cerebellar ataxia manifests in a vast majority of MERRF patients (approximately 40%) and is included in canonical criteria for clinical diagnosis [39,43]. Interestingly, myoclonic ataxia has been reported to be a possible better MERRF-defining feature than myoclonic epilepsy [39]. Ragged red fibres (RRFs) in muscle biopsy, one of the traditional characteristics, may occasionally not be observed [43]. The most commonly detected mtDNA mutation in MERRF patients is an adenine to guanine mutation at nt8344 in the tRNALys gene (*MT-TK*). This point mutation causes a low aminoacylation of the mutant tRNA, as well as hypomodification of its anticodon loop and incomplete translation at several lysine codons, leading to decreased mitochondrial membrane potential and lower electron flux through the transport chain [5,44]. Recently, direct reprogramming of MERRF patient-derived fibroblasts into induced neurons was achieved, allowing the observation of patterns of altered mitochondrial dynamics, pathological activation of the mitophagy pathway and ROS overproduction [44]. Four *MT-TK* pathogenic variants account for approximately 90% of cases. In less than 5% of cases, mutations in other mitochondrial tRNA encoding genes (*MT-TF*, *MT-TH*, *MT-T1*, *MT-TL1*, *MT-TP*, *MT-TS1*, *MT-TS2*) have been documented. Mutations in *MT-TK* and *MT-TL1* genes have been reported in MERRF/MELAS and MERRF/Kearns-Sayre syndrome overlap syndromes [45,46]; moreover m.8344A > G mutation has also been observed in LS, isolated myoclonus, isolated myopathy and multiple symmetric lipomatosis, in which ataxia has been reported in some cases [5,47]. Neuropathologic changes include cerebellum (dentate nucleus and cortex) and posterior spinal cord neuronal and fibres loss, together with brainstem (pontine tegmentum, inferior olivary nucleus) and basal ganglia degeneration [48,49,50].

#### 5.1.2. Neuropathy, Ataxia and Retinitis Pigmentosa (NARP)

NARP is characterised by proximal neurogenic muscle weakness with sensory neuropathy, ataxia and pigmentary retinopathy [51]. The onset of symptoms is typically in childhood, presenting with ataxia and learning difficulties; however, visual symptoms may be the only clinical manifestation. Prior to the onset of visual field constriction, ophthalmoscopy may reveal salt-and-pepper retinopathy [52]. NARP progression is usually slow; nevertheless, patients may experience episodic deterioration, often associated with virosis [53]. Ataxia is traditionally considered mainly sensory, due to the sensory (in some cases sensorimotor) polyneuropathy [51]. Genetically, NARP and maternally inherited Leigh syndrome (MILS, later described) are part of a continuum of progressive neurodegenerative disorders, caused by mitochondrial energy production abnormalities. In particular, NARP is most frequently caused by heteroplasmic point mutations in *MT-ATP6*, an mtDNA gene encoding subunit 6 of mitochondrial H (+)-ATPase (complex V); 70–90% of mutation loads usually manifest as NARP syndrome; meanwhile, higher levels of mutants may lead to MILS [54,55]. The most common pathogenic variants in *MT-ATP6*, m.8993T > G and m.8993T > C, show the strongest genotype-phenotype correlation of any mtDNA mutations described [56]. Pathogenic variants in *MT-ND6* and *MT-TV*, encoding a complex I subunit and tRNAVal, respectively, have also been reported in NARP patients [57]. Experiments on human fibroblast harbouring the NARP-associated m.8933T > G mutation indicate that complex V deficiency could be rescued by increasing mitochondrial substrate-level phosphorylation through alpha-ketoglutarate/aspartate supplementation, adding useful information to the current knowledge of NARP and MILS pathogenesis [58]. In patients with a high degree of mutant heteroplasmy, CNS pathological abnormalities in the cerebellum and basal ganglia may contribute to an ataxia phenotype [59]. A recent case report in a genetically confirmed NARP patient accounted peculiar histopathological features of the sural nerve, such as endoneurial accumulation of Schwann cell nuclei, clusters of concentric Schwann cell processes lacking myelinated axons. Mitochondrial ultrastructural abnormalities were detected in neurons, Schwann cells and muscle fibres [53].

#### 5.1.3. Maternally Inherited Leigh Syndrome (MILS)

Leigh syndrome (or subacute necrotizing encephalomyelopathy) is a clinically and genetically heterogeneous disease: more than 90 monogenic causes, either mitochondrial and nuclear, have been recognized [60]. We will now review the mtDNA-related Leigh syndrome or maternally inherited Leigh syndrome (MILS), in which cerebellar ataxia is present in nearly all patients. In MILS, onset of symptoms typically occurs in healthy children before the age of three, often following a viral infection or other metabolic stressors. Adult onset is infrequent. Neurologic features at onset include hypotonia, spasticity, cerebellar ataxia, chorea and peripheral neuropathy. Note that in the presence of severe hypotonia, ataxia may be masked. MILS may also present as a multisystem disorder. About 50% of affected patients die by the age of three, typically as a result of cardio-respiratory failure [60]. Decompensation during an intercurrent illness is typically associated with psychomotor retardation/regression, neurological decline and the development of a peculiar brainstem and basal ganglia bilateral symmetrical lesions with an elevated lactate level in spectroscopy. In patients with atypical neuroradiological findings, a diagnosis of Leigh-like syndrome can be considered [60,61]. As mentioned above, the most common genetic causes of MILS are pathogenic variants in *MT-ATP6* (m.8993T > G is the most common): the mutation load correlates with the phenotype severity. Other frequently reported affected mitochondrial genes are: *MT-ND3*, *MT-ND5* (m.13513G > A is the most common variant) and *MT-ND6* (m.14487T > C is the most common variant), encoding three different subunits of NADH-ubiquinone oxidoreductase (complex I). Less common mtDNA mutated genes are *MT-CO3* and MT-TK, encoding a complex IV subunit and tRNALys, respectively [60]. The severe ATP depletion caused by the mitochondrial energy production impairment, together with gliosis, hyperlacticacidemia, ROS over-production and excitotoxicity, more than likely jointly contribute to the neurodegeneration observed in MILS. Interestingly, neuroimaging findings of dominant cerebellar edematous changes with petechial components suggestive of microangiopathy, have been recently reported in a MILS child. These findings support the hypothesis of a possible contribution of microvascular impairment in PMDs’ pathogenesis [40,62]. Typical neuropathologic changes are multiple focal symmetric necrotic lesions with a spongiform appearance in the basal ganglia, thalamus, brain stem, dentate nuclei and optic nerves. Histologically, these lesions are marked by gliosis, demyelination and vascular proliferation [60].

#### 5.1.4. Mitochondrial Encephalomyopathy, Lactic Acidosis and Stroke-like Episodes (MELAS)

MELAS is a multisystem disorder with protean manifestation, generally presenting between the ages of two and forty years. MELAS is defined by the presence of three major features: (1) stroke-like episodes, due to focal, often asymmetric brain lesions not corresponding to well-defined vascular territories. They are usually localized in the temporal, parietal and occipital cortical or subcortical areas and show decreased N-acetylaspartate signals and peak of lactate at spectroscopy [63,64,65] (2) encephalopathy, marked by seizure and/or dementia and (3) myopathy, evident by lactic acidemia and RRFs on muscle biopsy [5]. Frequent initial symptoms are focal or generalized seizures, recurrent headaches, stroke-like episodes, cortical vision loss, muscle weakness and recurrent vomiting. Cerebellar ataxia, as an additional clinical manifestation, can be observed in 25–49% of MELAS patients. As LS, MELAS frequently has a multisystem involvement [63]. The most common pathogenic variant associated with MELAS is m.3243A > G in the *MT-L1* gene, encoding tRNALeu. Additional mutations (e.g., m.3271T > C and m.3252A > G) in the *MT-TL1* gene can also cause MELAS. Rarely, mutations in other tRNA genes, such as *MT-TL2*, *MT-TK*, *MT-TH*, *MT-TQ*, *MT-TF* and *MT-TV*, as well as in respiration complex subunits encoding genes, have been reported. Pathogenic mutations in nuclear genes, such as *POLG1*, have also been associated with mitochondrial encephalopathy [66]. Defects in the tRNA genes decrease mitochondrial protein synthesis leading to impaired energy production, that in turn, stimulate mitochondrial proliferation, increase reactive nitrogen and oxygen species production and cause nitric oxide deficiency. The result is a microvascular blood perfusion impairment, which significantly contributes to the multi-organ dysfunction observed in MELAS, particularly to stroke-like episodes. Moreover, stroke-like episodes may not have a vascular aetiology but be primitively metabolic, due to transient OXPHOS dysfunction within the brain parenchyma. It is important to recall that the m.3243A > G mtDNA mutation is associated with a heterogeneous spectrum of phenotypes, ranging from the most severe MELAS syndrome to asymptomatic carrier; going through this phenotypic spectrum, intermediate single-organ (e.g., cardiomyopathy, diabetes) or multi-organ involvement (maternally inherited deafness and diabetes or MIDD, chronic PEO or cPEO, MERRF, MILS or non-syndromic phenotypes) may be present. The nuclear genetic background may influence this clinical heterogeneity in m.3243A > G-related disease [66]. Interestingly, slow progressive cerebellar ataxia has been reported in single cases of MIDD, associated with the m.3243A > G mtDNA mutation [67,68]. Neuropathologically, as for cerebral white matter, cerebellar fibres damaged by stroke-like episodes show diffuse fibrillary gliosis, apart from cortical degeneration [5,63].

#### 5.1.5. Leber Hereditary Optic Neuropathy (LHON)

LHON is a blinding disorder, typically presenting as a bilateral, painless, subacute visual failure. Its onset is generally in the second or third decades of life, with males being up to four times more likely to be affected. LHON was the first disease to be associated with mtDNA point mutations, the most frequent PMD and the first for which a treatment has been approved [69]. Neurological supplementary features, including cerebellar ataxia, have been reported in a minority of LHON cases, configuring a phenotype known as ‘LHON-plus’ [70,71,72,73]. The most common cause of LHON, accounting for about 90% of cases, are mtDNA amino acid substitutions (11778A > G, m.14484T > C, m.3460G > A), usually homoplasmic, in *MT-ND4*, *MT-ND6*, *MT-ND1* genes, respectively. These genes encode different complex I subunits, the most frequently affected respiratory complex in the majority of LHON-associated mutations. The selective degeneration of retinal ganglion cells in LHON is the result of the global bioenergetic perturbation; complex I deficiency and ROS overproduction play major roles [69]. The incomplete penetrance of LHON-associated mtDNA point mutations and the prevalence in males are partially explainable considering the evidence of modifying factors such as nDNA mutations, secondary mtDNA mutations, mitochondrial haplogroups and environmental triggers [69]. Different combinations of mtDNA mutations, indeed, either heteroplasmic or homoplasmic, have been associated with the LHON-plus phenotype. In this case, optic atrophy is coupled with olivo-ponto-cerebellar projections degeneration, explaining the cerebellar ataxia manifestation [71].

### 5.2. Disorders of mtDNA Defects: Large Scale Rearrangements

#### Kearns-Sayre Syndrome (KSS)

KSS is a severe, usually sporadic, multisystem disorder defined by a clinical triad consisting of: onset before the age of twenty years, PEO and pigmentary retinopathy. At least one of the following additional features should be present: cardiac conduction defects leading to a complete heart block, cerebellar ataxia and/or elevated CSF protein level. Epilepsy and stroke-like episodes, compared to other mitochondrial encephalomyopathies, are rare. KSS usually progresses to death in early adulthood [74]. KSS, Pearson syndrome (PS) and cPEO are three overlapping phenotypes caused by large-scale rearrangement of mtDNA: deletions, ranging in size from 1.1 to 10 kb, duplications or a combination of both. A single large deletion of 4977 bp is the most common deletion associated with KSS; however, more than 150 different mtDNA deletions have been associated with it; these can be detected either in muscle tissue or blood cells [74]. mtDNA large-scale rearrangements usually occur de novo; however, offspring of a female proband have up to a 4% risk of being affected [18]. Together with large-scale mtDNA rearrangements, numerous pathogenic variants in either mtDNA and nDNA genes have been associated with cPEO; the identification of multiple mtDNA deletions must raise the suspicion of an underlying nDNA aetiology [2,74]. Ataxia is not a cardinal feature of cPEO and PS. Nevertheless, the three phenotypes can overlap and may evolve from one clinical syndrome to another in a given individual over time; for example, PS patients may even develop into KSS and LS and manifest cerebellar ataxia [60,74,75]. To further emphasize the fluid heterogeneity of mtDNA-related PMDs, the term “KSS spectrum” has been proposed. This phenotypic category includes classic KSS and cPEO with multisystem involvement [76]. KSS neuropathology is characterised by status spongiosus, with predominant white matter modifications in the cerebral and cerebellar compartment and grey matter degeneration in the brainstem. Spongiform degeneration and peculiar ‘mitochondrial angiopathy’ (marked by vascular proliferation) around the dentate nucleus are thought to be the driver of the loss of Purkinje cells and the disconnection of the cortico-nuclear output of the cerebellar system [50,77].

### 5.3. Disorders of nDNA Defects: Respiratory Chain Subunits, Ancillary Proteins, Electron Carriers

#### 5.3.1. Leigh Syndrome (LS)

Pathogenic mutations in nuclear genes encoding respiratory chain subunits (‘direct hits’) have been identified in all five complexes. Often, the resulting diseases manifest at birth or soon after, with remarkably homogeneous phenotypes resembling severe forms of LS and autosomal recessive inheritance patterns [3]. As previously discussed, LS clinical manifestations are dominated by CNS involvement but it may also have extra neurological manifestations [60]. Neuroradiological and neuropathological features, including brainstem and cerebellum involvement, approximate the one described for MILS, explaining the frequent occurrence of ataxia. However, isolated involvement of the cerebellum is uncommon [61]. Complex I subunits are the most frequently affected: mutations in *NDUFS4*, encoding Fe-sulfur protein 4 subunit, and less frequently, in complex I flavoprotein1 (*NDUFV1*) and Fe-sulfur protein 1 subunit (*NDUFS1*), are the most abundant [60]. Deficiencies of succinate dehydrogenase (complex II), encoded completely by nDNA genes, and ubiquinol-cytochrome c oxidoreductase (complex III) are rare causes of LS and collectively underlie <10% of all cases [60]. Ataxia is especially frequent in patients with biallelic *SDHA* pathogenic mutations, a complex II direct hit [78]. Finally, mutations in cytochrome c oxidase (complex IV) subunits, especially 6B1 and 7B, are extremely rare [60]. However, mutations in *SURF1*, encoding a complex IV assembly protein (‘indirect hits’, see below), underlie the largest fraction of complex IV-associated LS. As previously mentioned, truncal ataxia and intentional tremor are prominent neurological features, as well as brain MRI cerebellar and brainstem symmetric lesions [60].

#### 5.3.2. GRACILE Syndrome

Mutations in genes encoding ancillary proteins, required for respiratory chain protein transport and assembly, prosthetic group acquisition and multimerization are referred to as ‘indirect hits’. BCS1 is a mitochondrial chaperone needed for Rieske Fe-sulphur subunit insertion in complex III. Mutations in the BCS1-encoding gene cause GRACILE syndrome, characterised by growth retardation, aminoaciduria, cholestasis, iron overload and early death [3]. Ataxia may be present as an additional feature, in the context of a metabolic encephalopathy [79].

#### 5.3.3. Primary Coenzyme Q_10_ deficiency

The mitochondrial electron transport chain includes two mobile electron carriers, coenzyme Q (CoQ_10_) and cytochrome c (Cyt c) that shuttle electrons through respiratory complexes. CoQ_10_, or ubiquinone, carries electrons from complex I and II to complex III, also acting as an antioxidant and regulating the apoptosis intrinsic pathway [1]. Primary CoQ_10_ deficiency is a genetically heterogeneous disorder that, traditionally, has been thought to occur with five different clinical phenotypes: an isolated myopathic form, a form characterised by steroid-resistant nephrotic syndrome (without mutation of nephrin and podocin encoding-genes) accompanied by deafness, retinopathy and other CNS manifestations, a mitochondrial encephalomyopathy form resembling an LS or MELAS phenotype, a severe infantile multisystem form and a pure isolated ataxic form [80]. However, this classification is not sufficient to capture the complexity of this group of diseases, since different overlapping phenotypes have been identified. The ataxic form is the most common variant: cerebellar ataxia can be isolated or present with further manifestations such as epilepsy, migraine, muscle weakness and developmental delay. In clinical practice, it must be considered in the differential diagnosis of juvenile ataxias and infantile encephalomyopathies [80]. Pathogenic variants in 8 of the 13 genes involved in the CoQ_10_ biosynthetic pathway (*PDSS1*, *PDSS2*, *COQ2*, *COQ4*, *COQ6*, *ADCK3* or *COQ8A*, *COQ9*) have been ascribed to primary CoQ_10_ deficiency, usually having an autosomal recessive pattern of inheritance. In *COQ8A*-related disease, affected individuals experience onset of muscle weakness and exercise intolerance in early childhood, followed by cerebellar ataxia with severe cerebellar atrophy on MRI [81]. The disease course can be progressive or self-limited. Individuals with primary CoQ_10_ deficiency may respond well to high-dose oral CoQ_10_ supplementation; however, patients harbouring mutations in *COQ8A* typically benefit less from it [80,81].

### 5.4. Disorders of mtDNA Replication and Expression

Defects in the nuclear-encoded mitochondrial replisome result in diseases associated with multiple mtDNA deletions or mtDNA depletion. The replication machinery includes a polymerase enzyme, composed of a catalytic (POLG) and an accessory (POLG2) subunit, a replicative helicase (TWNK) and a helicase/nuclease (DNA2). Moreover, six enzymes control the subtle balance of the mitochondrial deoxyribonucleoside triphosphates pool, necessary for mtDNA replication.

#### 5.4.1. Infantile-Onset Spinocerebellar Ataxia (IOSCA)

IOSCA was originally described in individuals of Finnish descent, however non-founder variants associated with atypical-IOSCA have been described. Classic IOSCA is a progressive neurodegenerative disorder having onset in infancy and manifesting with severe spinocerebellar ataxia, profound muscle hypotonia, loss of deep-tendon reflexes, and athetosis. Typical late-manifesting features are axonal neuropathy, optic atrophy and hypergonadotropic hypogonadism (in females). Late-manifesting epilepsy may lead to encephalopathy. In contrast, atypical IOSCA is usually more rapid, severe and exhibits liver involvement. Liver tissue samples may show mtDNA depletion [3,82,83,84]. IOSCA is caused by *TWNK* (or *PEO1/C10orf2*) mutations which lead to an accumulation of multiple mtDNA deletions. Individuals who are homozygous for the founder variant (c. 1523A > G) show a classical IOSCA phenotype [84]. Atypical IOSCA, has been associated with compound heterozygous (c. [1523A > G] − [952G > A]) or c. 1370C > T homozygous conditions. Recently, a novel disease phenotype that retains the cardinal IOSCA features but also includes myopathy and mtDNA deletions in skeletal muscle was found in a Korean family [85]. Neuropathologically IOSCA is marked by aggressive spinocerebellar degeneration resulting in cortical, cerebellar and brainstem progressive atrophy.

#### 5.4.2. POLG1-Associated Mitochondrial Ataxias

Recessive mutations in *POLG1* have been associated with a heterogeneous spectrum of neurological and musculoskeletal disorders, all of which can have ataxia, either sensory, cerebellar or spinocerebellar, as cardinal or additional features [83]. This large spectrum includes childhood myo-cerebro-hepato syndromes, Alpers-Huttenlocher syndrome (AHS), ataxia neuropathy spectrum disorders (SANDO and MIRAS) and disorders with epilepsy, myopathy, and ataxia without ophthalmoplegia (MEMSA) [83,86]. Evidence of vast complex I defects affecting interneurons and Purkinje cells and extensive involvement of GABAergic neurons in post-mortem brains from patients with AHS has been discovered recently [87]. In addition, most of the rare cases of AR-CPEO are caused by *POLG1* mutations which may have ataxia as an ancillary feature. Dominant mutations in *POLG1* instead, are usually associated with adult-onset PEO phenotypes (AD-CPEO) and variable degrees of extra-ocular manifestations such as extrapyramidal signs, spinocerebellar ataxia, peripheral neuropathy, mental retardation, hypogonadism and gastrointestinal dysmotility (‘CPEO plus’) [83,86]. There are nearly 250 pathogenic mutations in *POLG1* affecting five distinct functional modules of the enzyme; in several instances, the same mutations have been reported in different syndromes, reinforcing the idea of a *POLG1*-related spectrum of diseases. The most recurrent mutations are two amino acid changes in the spacer region of the pol-gammaA protein (A467T and W738S) which result in insufficient polymerase activity along with compromised interaction with the accessory subunit and a severe DNA binding defect, respectively [88].

#### 5.4.3. Ataxia Neuropathy Spectrum (ANS)

ANS affects patients in their midteens and usually results in premature death. It includes mitochondrial autosomal recessive ataxia syndrome (MIRAS) and a different nosological entity known as sensory ataxia neuropathy dysarthria and ophthalmoplegia (SANDO) [89]. MIRAS is characterised by progressive cerebellar ataxia and neuropathy, either motor, sensory or mixed, which can, if severe, contribute to ataxia manifestations (‘spinocerebellar ataxia’). Some individuals may manifest slow progressive encephalopathy and hepatic failure, usually as a consequence of valproate administration. Clinical myopathy is rare, despite approximately 25% of individuals having cramps [86]. Migraine-like headaches may be present years before the onset of ataxia. Sensory ganglionopathy, speech disturbance and PEO, instead, characterise a SANDO phenotype. ANS should be considered as a first line-differential diagnosis of progressive ataxia syndromes in Europe, given the high frequency of mutations in the northern part of the continent [90]. Neuropathological findings include dorsal column atrophy, cortical cerebellar atrophy due to loss of Purkinje cells and deep cerebellar nuclei changes. No signs of vasculopathy, as for mtDNA-related disorders, have been described, proving that the thalamic and occipital lesions are not a consequence of ischemic episodes [90,91,92].

#### 5.4.4. Myoclonus Epilepsy, Mitochondrial Myopathy and Sensory Ataxia (MEMSA)

MEMSA, previously referred to as spinocerebellar ataxia with epilepsy (SCAE), is an overlapping spectrum of myopathy (with or without RRFs), epilepsy and ataxia in the absence of ophthalmoplegia. It generally manifests in young adulthood. A subclinical sensory polyneuropathy, leading to sensory ataxia, is usually the first sign, followed years later by local myoclonic seizures, frequently involving the right arm, which can generalise. Recurrent bouts of seizure activity are accompanied by progressive interictal encephalopathy. The myopathy may be distal or proximal and can be subclinical, presenting with exercise intolerance [83,86,93].

#### 5.4.5. Leukoencephalopathy with Brainstem and Spinal Cord Involvement and Lactoacidosis (LBSL)

mtRNA translation defects usually present in infancy with severe neurological involvement (LS, recessive ataxia, leukodystrophy) and/or cardiomyopathy, lactic acidosis and hepatocerebral syndrome. They are biochemically characterised by multiple respiratory complex deficiencies without evidence of mtDNA depletion or multiple deletions [3]. LBSL is an autosomal recessive genetic disorder consisting of slowly progressive cerebellar ataxia, spastic paraparesis and posterior cord syndrome occurring generally in childhood or adolescence after a normal early development. Dysarthria develops over time and deep tendon reflexes are usually retained. Few cases of antenatal, early-infantile onset or adulthood onset LBSL have been reported [94,95]. To date, more than 100 individuals have been identified with biallelic pathogenic variants in *DARS2*, encoding mitochondrial aspartyl-tRNA synthetase. Eight-eight per cent of LBSL patients carry a mutation in the intron 2 splice acceptor region. Despite mitochondrial aspartyl-tRNA synthetase being a ubiquitously expressed enzyme, *DARS2* mutations particularly affect the nervous system [95]. LBSL pathogenesis, largely still under study, has its primary driver in aminoacylation impairment. Nonetheless, secondary aspartyl-tRNA synthetase functions, including inflammatory and immune regulation, have been hypothesised [95]. Fluctuant lactate elevation may be detected by MRI spectroscopy in most LBSL patients; however, it is not a diagnostic criterion. MRI, indeed, is the preliminary step for diagnosis; LBSL has three major neuroimaging diagnostic criteria: signal changes in the cerebral white matter, spinal cord dorsal columns and lateral corticospinal tract involvement, medulla pyramidal or medial lemniscal tract involvement. Genetic testing ultimately differentiates LBSL from other leukodystrophies [95].

### 5.5. Disorders of nDNA Defects: Mitochondrial Dynamics, Homeostasis and Quality Control

Interference with mitochondrial dynamics, morphology maintenance, fusion or fission results in disease. Given the importance of mitochondrial motility along neuronal axons and their dependence on OXPHOS, CNS is particularly vulnerable.

#### Autosomal Dominant Optic Atrophy Syndrome (ADOA)

Cerebellar ataxia may be an additional manifestation of ADOA syndrome in the context of the so-called ‘ADOA plus’, accounting for approximately 20% of all ADOA cases [69]. ADOA plus is marked by bilateral and symmetric progressive visual loss occurring typically during the first decade of life. Subsequently, other manifestations may appear, including ataxia and axonal sensory-motor polyneuropathy [69]. The most frequent genetic causes are heterozygous mutations in the *OPA1* gene, encoding a dynamin-like GTPase involved in mitochondrial fusion, cristae organisation, energy production and apoptosis. ADOA patients may also harbour multiple mtDNA deletions suggesting a role of *OPA1* in mtDNA stability maintenance [69]. A few additional cases have been attributed to mutations in other genes. The disease is transmitted in an autosomal dominant way; however, some *OPA1* mutations also appear to exhibit incomplete penetrance. Moreover, *OPA1* has been implicated also in biallelic forms of syndromic optic atrophy, such as Behr syndrome, which exhibits extra-ocular manifestation including cerebellar ataxia [69]. Both patient fibroblasts and cell models bearing pathogenic mutations of *OPA1* have a characteristic fragmented mitochondrial network, highly disordered cristae and OXPHOS dysfunctioning due to the role of OPA1 in the stability of respiratory super-complexes [96].

### 5.6. Disorders of nDNA Defects: Metabolism of Substrates

Diseases associated with genes involved in the Krebs cycle or pyruvate metabolism are commonly considered as PMDs, despite the latter not causing classic OXPHOS dysfunction. Fatty acid oxidation, ketone bodies metabolism or anaplerotic pathway genetic disturbances instead, are controversially regarded as causes of PMDs.

#### Pyruvate-Dehydrogenase Complex-Deficiency

Pyruvate dehydrogenase complex (PDC) deficiency is an inborn error of mitochondrial energy metabolism. PDC, comprising three enzymes and one structural component, decarboxylates pyruvate to acetyl-CoA, bridging the glycolytic pathway in the cytosol to the mitochondrial Krebs cycle [1]. According to the severity, the heterogeneous spectrum of clinical phenotypes can be divided into three categories: neonatal, presenting with fatal lactic acidosis and cardio-vascular insufficiency; infantile, presenting by progressive encephalomyopathy leading to LS; benign, usually characterised by chronic neurological dysfunctions [97]. The majority of affected individuals harbour mutations in the X-linked *PDHA1* gene, encoding the E1α subunit of PDC. The remaining cases are caused by mutations in *PDHB* (encoding E1β) and other PDC subunit-encoding genes [97]. The wide spectrum of PDC disorder phenotypes includes recurrent episodes of isolated acute ataxia, usually triggered by stressors (i.e., virosis, fever), with complete recovery between episodes. During episodes, affected individuals may present signs of basal ganglia and brainstem dysfunctioning such as dysarthria, dystonia, choreoathetotic movements and hyperventilation [98]. Ataxia is usually less severe in patients carrying *PDHB* mutations.

### 5.7. Disorders of nDNA Defects: Metabolism of Cofactors

Dysfunctional OXPHOS due to cofactor deficiencies (i.e., heme A, CoA, NADPH, riboflavin, thiamine, biotin) are generally regarded as a cause of PMDs. *COX10* and *COX15* encode complex IV assembly factors and are required to farnesylate heme B to heme O in the heme A biosynthetic pathway. Mutations in these genes have been reported in both infants and adults. None of the infants survived beyond two years with some of them having LS and manifesting ataxia [99]. Of note, adult patients display a milder phenotype without cerebellar involvement [100].

## 6. Discussion and Conclusions

**Figure 1:**
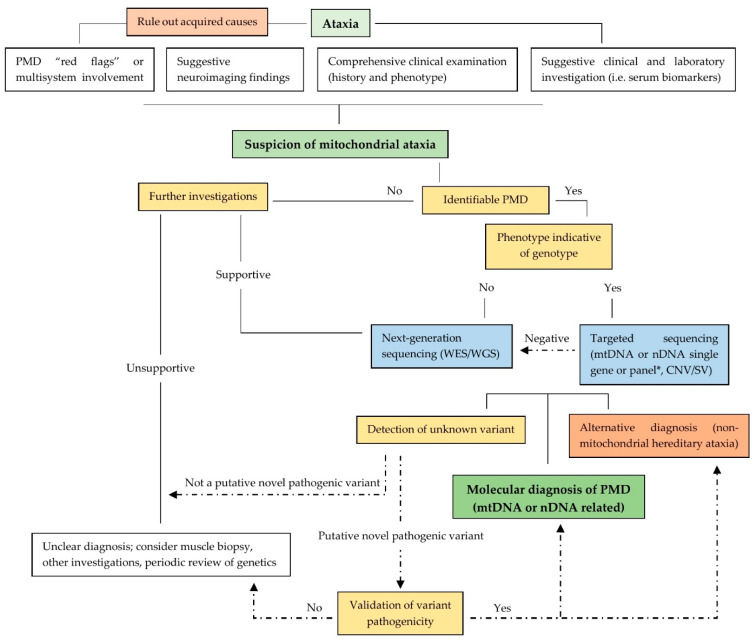
Diagnostic algorithm for mitochondrial ataxias. CNV/SV: copy number variants/structural variations. * In the case of a dominant family history, firstly screen for SCA 1, 2, 3, 6, 8, 17 and dentatorubral-pallidoluysian atrophy. In the case of a suspected recessive disease firstly screen for FRDA, ataxia-telangiectasia, spastic ataxias, *POLG1* mutations and oculomotor apraxia type 1 and 2.

The profound dependency of CNS on aerobic metabolism explains the high frequency of neurological manifestation in PMDs. Among these, ataxia, either sensory or cerebellar, is part of the clinical spectrum of PMDs in both paediatric and adult patients. In Figure 1, we propose a diagnostic algorithm for mitochondrial ataxias. The first section shows clues that can help raise suspicion of mitochondrial ataxia. After that the algorithm integrates the diagnostic approaches of PMDs and hereditary ataxias (see above paragraphs). Finally, it shows the pathway for the validation of unknown variants’ pathogenicity.

Ataxia in PMDs can be present in the context of complex multisystem phenotypes, either syndromic and non-syndromic or, sometimes, as a prominent feature. It is often cerebellar, usually presenting with a progressive pattern of evolution. Sensory ataxia is less common and may suggest a specific syndrome such as *POLG1*-related disease or NARP.

No specific findings define mitochondrial ataxia, but its co-occurrence with neurological and multisystem non-neurological peculiar signs or symptoms (Table 1) must raise the suspicion of PMD.

Neuroimaging findings can support diagnosis: along with cerebellar atrophy, peculiar alterations should be searched (Table 4). T2/FLAIR hyperintense, bilateral, and symmetric white-matter alterations in cerebellar and cerebral hemispheres have been described in *POLG1*-related ataxias, MERRF and KSS patients [22]. Interestingly, cerebellar hemispheres’ involvement may be detected years before the development of stroke-like episodes in MELAS [64,65]. Moreover, cerebellar hemispheric lesions resembling infarcts may be seen in *POLG-1* related ataxias [91]. Deep cortical, brainstem and cerebellar atrophy is detected in IOSCA [23]. Within the cerebellum, the dentate nuclei and the dentatorubral fibres, crossing the superior cerebellar peduncle, are frequently severely affected in KSS [22]. In the brainstem, the midbrain may present signal alteration in KSS patients; inferior olivary nuclei lesions should prompt a search for *POL*G1 mutations [22,91]. Basal ganglia signal changes, especially in the dorso-medial thalamic region, are peculiar findings in KSS and *POLG1*-related phenotypes [22,91]. More recently, spinal cord white and grey matter involvement in KSS has been described [101]. LS, MELAS and LBSL have distinctive MRI patterns that we describe in Table 4.

A detailed family history should be collected. The maternal inheritance pattern is the cardinal clue for the diagnosis of an mtDNA-related PMD, however, low heteroplasmy in pedigree members may complicate the clinical scenario. In the case of a recessive pattern of inheritance instead, *POLG1*-related disorders should be included in the first-line genetic screening for hereditary ataxia. Nonetheless, pathogenic variants in both mtDNA and nDNA can occur de novo, manifesting as sporadic PMDs.

Mitochondrial ataxias should be included in the differential diagnosis of hereditary ataxias; a meticulous clinical evaluation of affected patients should guide the genetic testing to untangle the complexity of the molecular background and reach a diagnosis.

## Figures and Tables

**Table 1:** Phenotypic expression of PMDs.

Tissue/System	Sign or Symptom
*Neurological*	
CNS	Epilepsy
	Ataxia
	Myoclonus
	Stroke
	*Stroke-like episode*
	Myelopathy
	Cortical blindness
	Migraine-like headache
	Psychomotor retardation/regression
	Encephalopathy/coma
	Dystonia
	Parkinsonism
	Cognitive impairment/Dementia
	Psychiatric disorders
PNS	Peripheral sensory-motor neuropathy
Muscle	Myopathy
	Exercise intolerance
Eye	*Progressive external ophthalmoplegia*
	Ptosis
	Retinitis pigmentosa
	Optic atrophy
	Cataracts
ENT	Sensorineural hearing loss
*Non neurological*	
Blood	Sideroblastic anaemia
	Bone marrow failure
Endocrine/reproductive system	Diabetes mellitus
	Short stature
	Hypoparathyroidism
	Multiple endocrinopathy
	Infertility
	Pregnancy loss
Heart	Cardiomyopathy
	Cardiac conduction defects
	*Wolff-Parkinson-White syndrome*
Liver and gastro-intestinal	Hepatopathy
	*Liver failure precipitated by valproic acid*
	Exocrine pancreas dysfunction
	*Intestinal pseudo-obstruction*
	Gastro-intestinal dysmotility
Kidney	Fanconi syndrome
	Renal tubular acidosis
	Focal segmental glomerulosclerosis
	Renal failure
	*Myoglobinuria*
Metabolism	Metabolic acidosis
	*Lactic acidosis*

In italics: peculiar signs/symptoms (‘red flags’) suggestive of PMDs.

**Table 2:** Ataxia in mtDNA-related PMDs.

Syndrome	Most Common Type of Ataxia		
	Cerebellar	Sensory	Spinocerebellar
*mtDNA point mutations*			
MERRF	●		
NARP		●	
MELAS	●		
LHON (plus)	●		
MIDD (not common)		●	
Multiple symmetric lipomatosis (not common)	●		
*mtDNA large scale rearrangements*			
KSS	●		
cPEO (not common)	●		
PS (not common)	●		

mtDNA-related PMDs in which ataxia can occur as rare additional feature are labelled as ‘not common’. In LHON, ataxia may manifest in ‘plus’ phenotype.

**Table 3:** Ataxia in nDNA-related PMDs.

Syndrome	Inheritance	Most Common Type of Ataxia		
		Cerebellar	Sensory	Spinocerebellar
*Respiratory chain subunits, ancillary proteins and electron carriers*				
LS	AR, AD			
Primary *CoQ10* deficiency	AR	●		
GRACILE (not common)	AR	●		
*mtDNA replication and expression*				
IOSCA	AR			●
MEMSA	AR		●	
MIRAS	AD			●
SANDO	AR			●
AHS	AR			●
AD-cPEO (not common)	AD			●
AR-cPEO (not common)	AR	●		
Mitochondrial neurogastrointestinal Encephalomyopathy (not common)	AR		●	
LBSL	AR	●		
*Mitochondrial dynamics, homeostasis and quality control*				
ADOA (plus)	AD		●	
3-methylglutaconic aciduria type III (not common)	AR	●		
3-methylglutaconic aciduria type V (not common)	AR	●		
*Metabolism of substrates*				
PDC-deficiency	XL, AR	●		
*Metabolism of cofactors*				
COX10-15 mutations (not common)	AR	●		
*Others*				
FRDA	AR		●	
XLSA/A	XL	●		
Wolfram syndrome (not common)	AR		●	

nDNA-related PMDs in which ataxia can occur as rare additional feature are labelled as ‘not common’. In ADOA, ataxia may manifest in ‘plus’ phenotype.

**Table 4:** Neuroimaging alterations suggestive of mitochondrial ataxia and specific PMDs.

Type of MRI Alteration	Suggested PMDs	References
Cerebellar atrophy	Not specific	[22,48,59,65,69,72,81,91,97]
T2/FLAIR hyperintense white matter	KSS, POLG1-related, MERRF, MELAS, IOSCA, ADOA plus	[22,23,48,65,69,91]
Dentate nuclei signal changes	KSS	[22]
Brainstem signal changes/atrophy	KSS, IOSCA	[22,23]
Inferior olivary nuclei lesions	POLG1-related	[91]
Basal ganglia lesions	KSS, POLG1-related, MERRF, NARP	[22,48,59,91]
Spinal cord signal changes	KSS	[101]
Cortical atrophy	IOSCA	[23]
*Brainstem and basal ganglia bilateral symmetrical lesions*	LS	[60,61]
*Stroke like lesions*	MELAS	[64,65]
*Cerebral white matter + dorsal column/lateral corticospinal tracts + pyramids*	LBSL	[95]

In italics: peculiar MRI patterns of LS, MELAS and LBSL.

## Data Availability

Not applicable.
